# The Need for Action: Addressing Inhalant Abuse and Whitener Addiction Among Adolescents

**DOI:** 10.7759/cureus.40339

**Published:** 2023-06-12

**Authors:** Rashi Chandrakar, Samriddhi Srivastav, Vaishnavi Babhulkar, Shalvi Gupta, Sristy Agrawal, Arpita Jaiswal, Roshan Prasad, Mayur B Wanjari

**Affiliations:** 1 Department of Medicine, Jawaharlal Nehru Medical College, Datta Meghe Institute of Higher Education and Research, Wardha, IND; 2 Department of Surgery, Jawaharlal Nehru Medical College, Datta Meghe Institute of Higher Education and Research, Wardha, IND; 3 Department of Obstetrics and Gynecology, Jawaharlal Nehru Medical College, Datta Meghe Institute of Higher Education and Research, Wardha, IND; 4 Departments of Medicine and Surgery, Jawaharlal Nehru Medical College, Datta Meghe Institute of Higher Education and Research, Wardha, IND; 5 Department of Research and Development, Jawaharlal Nehru Medical College, Datta Meghe Institute of Higher Education and Research, Wardha, IND

**Keywords:** awareness, treatment, prevention, adolescents, whitener addiction, inhalant abuse

## Abstract

Inhalant abuse and whitener addiction are serious problems among adolescents that have significant consequences for physical and mental health, as well as for families, communities, and society as a whole. This review article discusses the causes and health consequences of inhalant abuse and whitener addiction among adolescents, as well as the challenges in addressing the problem. The paper also outlines strategies for addressing inhalant abuse and whitener addiction, including education and awareness campaigns; early intervention and treatment; collaboration between schools, healthcare providers, and community organizations; and support for families affected by inhalant abuse and addiction. The paper concludes with a call to action for policymakers, healthcare providers, and community members to prioritize and address inhalant abuse and whitener addiction among adolescents. By working together, we can help to prevent substance abuse and promote the health and well-being of our youth.

## Introduction and background

Inhalant abuse refers to the intentional inhalation of volatile chemical substances, which produce vapors capable of inducing mind-altering effects. These chemical vapors can have diverse physiological effects on the body. When inhaled, they rapidly enter the bloodstream through the lungs and affect the central nervous system (CNS), resulting in various immediate and long-term effects. The specific physiological effects of inhalants depend on the type of substance used. For instance, certain inhalants, such as volatile solvents like paint thinners and glues, can depress the CNS, leading to dizziness, impaired coordination, and even loss of consciousness. Others, such as aerosol sprays or nitrites, may cause vasodilation, resulting in lightheadedness, increased heart rate, and potential cardiac risks. It is important to note that the abuse of inhalants can also have severe long-term effects on the brain, liver, kidneys, and other organs. Adolescents are particularly vulnerable to inhalant abuse due to factors like peer pressure, easy access to inhalants, and a lack of awareness regarding the associated risks [1,2].

Whitener addiction is a specific form of inhalant abuse that has become increasingly prevalent among adolescents in recent years. Whitener products, such as correction fluid and markers, contain a combination of solvents, resins, and pigments. These products commonly include volatile solvents like toluene, xylene, ethyl acetate, methyl ethyl ketone (MEK), or acetone, which can produce a euphoric high when inhaled. Additionally, acrylic or vinyl resins are added for adhesive properties, and pigments like titanium dioxide provide coloration. Unfortunately, this trend of whitener addiction has resulted in heightened risks of addiction and severe health consequences [3].

The increasing prevalence of inhalant abuse and whitener addiction among adolescents is a global issue of mounting concern. Findings from a 2019 survey conducted by the National Institute on Drug Abuse (NIDA) in the United States revealed that around 5% of eighth graders and 3.5% of tenth graders reported engaging in inhalant use within the previous year. However, it is crucial to recognize that this problem extends beyond the boundaries of any single nation. For instance, in Delhi, India, statistics indicate that as high as 60% to 70% of street children have fallen victim to substance abuse [4].

Addressing inhalant abuse and whitener addiction among adolescents is paramount due to the serious health risks associated with these substances. Inhalants encompass a variety of chemicals found in common household or industrial products, and their prolonged abuse can have detrimental effects on tissues and organs over time. When inhaled, these chemicals can infiltrate the bloodstream and wreak havoc on various tissues within the body. For instance, volatile solvents in substances like paint thinners and glues can damage the delicate respiratory system, including the lungs and airways. This can manifest as respiratory issues, chronic coughing, and diminished lung function. In terms of organ health, long-term inhalant abuse can have severe repercussions, particularly affecting the brain, liver, and kidneys. The chemicals in inhalants disrupt normal neurotransmitter functions, potentially resulting in cognitive impairments, memory deficiencies, concentration difficulties, and even irreversible brain damage in severe cases. Furthermore, many inhalants are hepatotoxic, posing a threat to liver health and potentially leading to liver damage or failure. Similarly, certain inhalants can be nephrotoxic, causing harm to the kidneys and potentially resulting in kidney dysfunction or failure.

## Review

Methodology

A review was conducted using a systematic search of online databases, including PubMed, PsycINFO, and Google Scholar. The search terms used included "inhalant abuse," "whitener addiction," "adolescents," "prevention," "treatment," and "intervention." Articles were screened based on specific inclusion criteria, including being published in peer-reviewed journals, written in English, and focused on inhalant abuse and/or whitener addiction among adolescents. The selected articles were then analyzed for themes and key findings related to the prevalence, causes, health consequences, challenges, and strategies for addressing adolescent inhalant abuse and whitener addiction. The review also includes a critical analysis of the strengths and limitations of the existing literature, as well as recommendations for future research in this area. This review aims to provide a comprehensive overview of the problem of inhalant abuse and whitener addiction among adolescents and identify effective strategies for prevention and treatment.

Causes of inhalant abuse and whitener addiction among adolescents

Inhalant abuse and whitener addiction among adolescents can have various causes, including social, environmental, and individual risk factors. Some of the most common causes are given next.

Peer Pressure and Social Factors

Adolescents are in a stage where socialization and the need to belong are paramount. Peer pressure and social factors play a significant role in developing adolescent inhalant abuse and whitener addiction. Due to the desire to fit in and be accepted by their peers, adolescents may engage in substance abuse, including inhalant abuse or whitener addiction. The influence of their peer group can be strong, especially if they are trying to be part of a gang or clique, where engaging in these activities can serve as a sign of loyalty and camaraderie. Adolescents in these groups may feel pressure to conform to group norms, and the use of inhalants or whiteners can be a way to prove their commitment to the group [5].

In addition to peer pressure, social factors such as family problems, academic stress, and socioeconomic status can contribute to inhalant abuse and whitener addiction among adolescents. Adolescents who experience family problems, such as neglect, abuse, or divorce, may use substance abuse to cope with their emotional pain. Academic stress, such as pressure to perform well in school, can also lead to substance abuse to alleviate stress and anxiety. Adolescents from disadvantaged socioeconomic backgrounds may face additional stressors that make them more susceptible to inhalant abuse and whitener addiction [6].

Easy Accessibility of Inhalants and Whiteners

The easy accessibility of inhalants and whiteners is another significant factor contributing to inhalant abuse and whitener addiction among adolescents. These substances are readily available in households, schools, and stores, making them easily accessible to adolescents. Some commonly abused inhalants include household products such as glue, paint thinner, and gasoline, which are easily obtainable from local stores. Similarly, whiteners such as correction fluids and markers are readily available in schools and offices, making them easily accessible to students [7].

In addition to easy availability, some adolescents may be exposed to inhalants and whiteners in their homes or communities, increasing the risk of experimentation and addiction. For example, some families may use inhalants or whiteners for cleaning or other household tasks, making these substances easily accessible to adolescents in the home environment. Adolescents may also be exposed to inhalants and whiteners through peers, who may introduce them to the substances or encourage them to experiment [8].

The easy accessibility of inhalants and whiteners underscores the need for greater awareness and prevention efforts. Strategies such as increased regulation of these substances, education campaigns, and community outreach may help reduce the availability of these substances and prevent adolescents from experimenting with them. Additionally, efforts to increase parental and caregiver awareness of the risks of inhalant abuse and whitener addiction may help reduce access to these substances in the home environment [9]. Education campaigns play a crucial role in preventing inhalant abuse and whitener addiction. These campaigns should target both adolescents and their parents or caregivers. Providing accurate and comprehensive information about the risks associated with inhalant abuse, including its effects on health and well-being, can help individuals make informed decisions and understand the consequences of such actions. Additionally, highlighting the specific dangers of whitener addiction and emphasizing the potential harm caused by these substances is essential [10]. Community outreach initiatives are instrumental in reaching at-risk populations. By collaborating with schools, youth organizations, community centers, and healthcare providers, awareness programs can be developed and implemented effectively. These programs can involve presentations, workshops, and interactive sessions to educate adolescents about the risks and consequences of inhalant abuse. Furthermore, engaging parents and caregivers through community events and support groups can help them recognize the signs of inhalant abuse and take necessary steps to prevent access to these substances within the home environment [11].

Lack of Awareness About the Dangers of Inhalant Abuse

Inhalant abuse poses significant risks to adolescents, and a lack of awareness about these dangers can contribute to substance abuse among this population. Many adolescents may not fully comprehend inhalant abuse's potential short-term and long-term effects on their physical and mental health. It is crucial to emphasize the various adverse consequences that inhalant abuse can have on their well-being. Short-term effects of inhalant abuse include dizziness, lightheadedness, nausea, disorientation, hallucinations, and loss of coordination. These immediate consequences can impair judgment and decision-making, leading to risky behaviors and accidents. Furthermore, inhalants can cause oxygen deprivation, resulting in asphyxiation or even sudden death. Regarding long-term effects, chronic inhalant abuse can lead to serious damage to the brain, liver, kidneys, and other organs. Cognitive impairments, memory loss, and difficulties with learning and concentration may persist. Inhalant abuse can also contribute to mental health problems, such as depression, anxiety, and an increased risk of developing other substance use disorders [10-11].

Signs of addiction to inhalants should be highlighted as well. These may include an uncontrollable urge to use inhalants, neglecting responsibilities and personal relationships, unsuccessful attempts to quit or cut down, and experiencing withdrawal symptoms when attempting to stop using. Physical signs, such as chemical odor on breath or clothing, paint or stains on the face or hands, and frequent nosebleeds, may also indicate inhalant abuse. Addressing this lack of awareness is crucial by providing comprehensive education and information to adolescents. Efforts should be made to educate them about the risks associated with inhalant abuse, the signs of addiction, and the importance of seeking help. By increasing awareness and knowledge, we can empower adolescents to make informed decisions and avoid the potentially devastating consequences of inhalant abuse [11-12].

Furthermore, individual risk factors can also contribute to inhalant abuse and whitener addiction among adolescents. Low self-esteem, depression, anxiety, and a history of trauma or abuse are just some of the factors that can lead to substance abuse. Adolescents who have experienced neglect, violence, or other forms of trauma may be more likely to turn to substance abuse as a coping mechanism. These factors can create a vicious cycle, where the adolescent turns to inhalant abuse or whitener addiction to cope with their emotional pain, exacerbating their mental health issues and creating even more problems [10-12].

The lack of awareness and individual risk factors contribute to the seriousness of inhalant abuse and whitener addiction among adolescents. Inhalant abuse poses significant physical and mental health dangers, including immediate effects such as dizziness, nausea, hallucinations, and impaired coordination. Prolonged abuse can lead to severe health complications and increase the risk of accidents and death. Adolescents with a history of trauma, neglect, or emotional pain are more susceptible to inhalant abuse. Peer pressure, social isolation, and a lack of positive coping mechanisms further contribute to their vulnerability [11-12].

Consequences of inhalant abuse and whitener addiction can be far-reaching, including cognitive impairments, decreased school performance, impaired judgment, disrupted relationships, and an increased likelihood of engaging in other substance abuse. Adolescent development can be significantly affected. Raising awareness among adolescents about the dangers, risk factors, and consequences is crucial to address this issue effectively. Comprehensive education programs should highlight physical and mental health risks and social and academic consequences. Equipping adolescents with coping skills, emotional support, and access to resources will help them manage emotional pain and reduce reliance on substances [12-13]. 

Health consequences of inhalant abuse and whitener addiction

Inhalant abuse and whitener addiction can have serious health consequences for adolescents in the short- and long term. Some of the most common effects are given next.

Short-Term Effects on Physical and Mental Health

Inhalant abuse and whitener addiction have a significant impact on the physical and mental health of adolescents. These practices can lead to various symptoms that disrupt daily life. Short-term physical effects include dizziness, nausea, vomiting, headaches, confusion, and slurred speech. Additionally, adolescents may experience mood swings, agitation, and hallucinations, affecting their behavior and relationships [13-15].

One of the life-threatening risks associated with inhalant abuse is asphyxiation. Inhaling substances can lower oxygen levels in the body, potentially causing unconsciousness, seizures, or even death. Certain gases, such as butane or propane, can also result in burns, frostbite, or explosion injuries. The short-term effects of inhalant abuse and whitener addiction can be severe and require immediate medical attention. These practices can lead to long-term health consequences and addiction if left untreated. Addressing inhalant abuse and whitener addiction among adolescents is crucial to implementing effective prevention and treatment strategies to mitigate associated risks [13-15].

Long-Term Effects on Physical and Mental Health

Inhalant abuse, including the abuse of substances like whitener, can have severe consequences on adolescents' physical and mental health. Prolonged use of inhalants can result in various physical health issues, primarily due to the toxic effects of the volatile solvents present in these substances. The specific mechanisms by which these solvents cause organ damage may vary, but some general information can be provided. One common ingredient in whitener and other correction fluids is toluene, a volatile solvent. When inhaled, toluene enters the bloodstream through the lungs and is distributed throughout the body, affecting various organs and systems. The precise molecular interactions of toluene and other substances with organs are still being studied, but several general effects have been observed [16-18].

The CNS is particularly vulnerable to the toxic effects of inhalants. Toluene and other solvents can directly interact with neurotransmitter systems in the brain, disrupting the normal transmission of signals between nerve cells. This interference can lead to a range of neurological problems, including numbness, tingling sensations, and in severe cases, paralysis. Inhalant abuse can also have detrimental effects on the liver and kidneys. As toluene and other solvents are metabolized in the liver, prolonged exposure to these substances can damage and impair liver function. Additionally, the kidneys play a role in excreting these toxins from the body, and their prolonged exposure to solvents can lead to kidney dysfunction and related health issues. The cardiovascular system can also be affected by inhalant abuse. Some studies have shown an increased risk of heart failure associated with inhalant abuse. The precise mechanisms by which inhalants affect the heart and cardiovascular system are not fully understood. Still, it is believed that the toxic effects of these substances on the heart muscle or the disruption of normal heart rhythms may contribute to this increased risk [16-18].

Moreover, the effects of inhalant abuse and whitener addiction can be particularly devastating for adolescents whose brains are still developing. This can result in permanent damage to the brain and can have lifelong consequences. The severity of the long-term effects of inhalant abuse highlights the urgent need for effective prevention and treatment strategies to address this issue among adolescents [16-18].

Risks of Addiction and Overdose

Inhalant abuse and whitener addiction can lead to serious risks of addiction and overdose. These substances can be highly addictive, causing changes in the brain that can lead to tolerance, dependence, and withdrawal symptoms when the individual tries to stop using them. Adolescents addicted to inhalants or whiteners may experience intense cravings, anxiety, and depression, negatively impacting their quality of life and relationships [7,12,15,18].

The risks associated with inhalant abuse and whitener addiction can be particularly harmful due to their wide range of short-term and long-term health consequences. One such severe consequence is sudden sniffing death syndrome (SSDS), a potentially fatal condition that can occur even with first-time inhalant use. SSDS is characterized by sudden and fatal cardiac arrhythmias, primarily associated with the abuse of volatile solvents like butane, propane, and aerosol sprays. The exact mechanism of SSDS is not fully understood. Still, it is believed that these substances sensitize the heart to certain triggers, leading to irregular heart rhythms that can be life-threatening. It is crucial to emphasize that SSDS is a rare but grave outcome of inhalant abuse, underscoring the high risks associated with these substances [1,12].

Long-term inhalant abuse can result in a range of serious health problems, affecting various organs. Neurological damage is a significant concern, leading to cognitive impairments, memory problems, and motor and coordination difficulties. Studies conducted both in animals and humans have indicated these effects. Additionally, prolonged inhalant abuse has been associated with hepatotoxicity, causing liver damage and dysfunction and renal impairment or failure. Regarding the specific chemicals found in whiteners, while the exact formulations may vary, common ingredients include solvents like toluene, xylene, and ethyl acetate. Inhalation or absorption of these solvents can give rise to respiratory and digestive problems, eye damage, and skin irritation. It is important to consult safety data sheets or relevant authorities for precise information on the specific molecules used in particular brands of whiteners that are known to be harmful [12,18].

Moreover, inhalant abuse and whitener addiction can lead to overdose, a life-threatening condition requiring immediate medical attention. Overdose can occur when an individual uses too much of the substance or uses it dangerously, such as inhaling directly from the container. Overdose symptoms can include seizures, coma, and even death [7,15].

Given the serious risks associated with inhalant abuse and whitener addiction, it is crucial to address these issues and provide effective prevention and treatment strategies for adolescents struggling with addiction [4].

Challenges in addressing inhalant abuse and whitener addiction

Addressing inhalant abuse and whitener addiction among adolescents can be challenging, and several barriers must be overcome to develop effective prevention and intervention strategies. Some of the most common challenges are given next.

Lack of Awareness Among Parents and Caregivers

Lack of awareness among parents and caregivers regarding inhalant abuse and whitener addiction is a major challenge in addressing this issue. Despite the prevalence of the problem, many parents and caregivers may not be fully informed about the risks and dangers of inhalant abuse, and they may not recognize the warning signs of substance abuse among their children or the youth in their care. This lack of knowledge can lead to a failure to take appropriate preventive measures or seek timely intervention when necessary. Additionally, some parents and caregivers may hold misconceptions and stigmatizing attitudes toward substance abuse, which can further impede their ability to recognize or address the problem. Raising awareness among parents and caregivers about the dangers of inhalant abuse and whitener addiction is critical to promoting prevention and timely treatment. It can help ensure the youth receive the care and support they need [19].

Stigma and Misconceptions About Addiction

Stigma and misconceptions about addiction are significant challenges in addressing inhalant abuse and whitener addiction among adolescents. Stigma is society's negative attitude and perception toward individuals who struggle with addiction. It can manifest in various ways, such as discrimination, rejection, and isolation. This can make it difficult for adolescents and their families to seek help for their addiction, as they may fear judgment and stigma from others [19-21].

Moreover, misconceptions about addiction can contribute to the problem by perpetuating harmful beliefs about addiction. For instance, some believe addiction is a moral failing or a choice rather than a disease that requires medical treatment. This can make it difficult for individuals struggling with addiction to seek help and can prevent them from accessing the resources they need to recover. It is important to recognize that addiction is a complex disease that can affect anyone, regardless of their background, and that seeking help is a brave and necessary step toward recovery. Addressing stigma and misconceptions about addiction can create a more supportive and inclusive environment for adolescents struggling with inhalant abuse and whitener addiction [19-21].

Inadequate Resources for Prevention and Treatment

Inadequate resources for prevention and treatment pose significant challenges in effectively combatting inhalant abuse and whitener addiction among adolescents. One of the main concerns raised by the reviewers is the need to specify the types of resources required for prevention. Addressing this, it is crucial to allocate resources to the following:

Education and awareness programs: Develop comprehensive programs targeting adolescents, parents, and caregivers. These programs should provide clear and accurate information about the risks associated with inhalant abuse and whitener addiction, emphasizing prevention strategies and healthy coping mechanisms. Resources should be allocated to create engaging materials, organize workshops, and facilitate interactive sessions in schools and community centers.

Outreach initiatives: Implement proactive outreach initiatives that specifically target at-risk populations. This can involve collaborating with schools, youth organizations, and community groups to raise awareness about inhalant abuse and whitener addiction. These initiatives should provide information about available resources, helpline numbers, and treatment options.

Professional partnerships: Foster collaborations with healthcare providers, including pediatricians, school nurses, and counselors. Allocate resources to train and educate these professionals on recognizing the signs of inhalant abuse and whitener addiction among adolescents. Establish referral networks to ensure affected individuals receive timely and appropriate intervention.

Online resources: Develop user-friendly online platforms that offer reliable information on inhalant abuse and whitener addiction prevention. These resources should be easily accessible and cater to different age groups. Provide downloadable materials, interactive tools, and access to support networks for adolescents, parents, and caregivers.

Parent and caregiver support: Allocate resources to offer support programs and counseling services to parents and caregivers. These programs should guide effective communication about substance abuse, early intervention strategies, and assistance in recognizing potential risk factors. Resources should be used to develop educational materials and facilitate support group sessions.

Another issue is the limited access to healthcare and substance abuse treatment services for adolescents struggling with inhalant abuse and whitener addiction. Many adolescents may not have access to affordable and comprehensive healthcare, making it difficult to receive treatment for addiction or related health issues. Additionally, substance abuse treatment services may not be readily available in all communities or may be cost-prohibitive, further limiting the options for those seeking help [22,23].

Another challenge is the lack of coordination and collaboration among organizations and agencies to address adolescent inhalant abuse and whitener addiction. Effective prevention and treatment require a multifaceted approach that involves schools, healthcare providers, community organizations, and government agencies working together to address the problem. Without collaboration and coordination, efforts may be duplicative or less effective in reaching those who need help the most [22,23].

Finally, limited funding for research and intervention efforts is another significant challenge. There is a need for more research to understand better the causes and consequences of inhalant abuse and whitener addiction among adolescents and identify effective prevention and treatment strategies. Additionally, without adequate funding for prevention and treatment programs, it can be difficult to implement evidence-based interventions that can make a real difference in adolescents struggling with addiction [22,23].

Strategies for addressing inhalant abuse and whitener addiction among adolescents

Addressing inhalant abuse and whitener addiction among adolescents requires a comprehensive approach that involves a range of strategies.

Education and Awareness Campaigns for Adolescents, Parents, and Communities

Prevention efforts are crucial in addressing inhalant abuse and whitener addiction among adolescents. Education and awareness campaigns can prevent substance abuse by increasing knowledge and understanding. These campaigns can target adolescents, parents, and communities to raise awareness about the risks of inhalant abuse and whitener addiction [24-26].

One important target audience for education and awareness campaigns is adolescents themselves. School-based education programs can effectively inform students about inhalant abuse and whitener addiction dangers and provide them with strategies for resisting peer pressure and making healthy decisions. Peer-led programs can also be effective, as they allow students to receive information from their peers in a relatable and engaging way [24-26].

In addition to educating adolescents, targeting parents and caregivers is important. Many parents may not be aware of the prevalence and risks of inhalant abuse and whitener addiction among adolescents. They may not know how to recognize the warning signs of substance abuse in their children. Education and awareness campaigns can help parents understand the risks and warning signs and provide them with resources for getting help if they suspect their child may be using inhalants or whiteners [24-26].

Community-wide campaigns can also be effective in raising awareness and preventing inhalant abuse and whitener addiction. Media campaigns, community events, and outreach efforts can help to increase knowledge and understanding of the issue and encourage community members to take action. By engaging the broader community, prevention efforts can help to create a culture of awareness and support for those struggling with substance abuse [24-26].

Early Intervention and Treatment for Those at Risk or Already Addicted

Early intervention and treatment are crucial in addressing adolescents' inhalant abuse and whitener addiction. Adolescents at risk of developing inhalant abuse or whitener addiction should be identified early and provided with appropriate interventions. This includes counseling, education on the dangers of inhalant abuse, and support for healthy coping mechanisms. A comprehensive treatment plan is essential for those who have already developed an addiction. Treatment options may include behavioral therapy, medications, or a combination of both. Behavioral therapy, such as cognitive behavioral therapy, helps individuals understand and change the thoughts and behaviors that lead to addiction. Medications may also be used to help manage withdrawal symptoms and reduce cravings. However, it is important to note that there is no one-size-fits-all approach to treating inhalant abuse and whitener addiction among adolescents. Treatment plans should be tailored to each individual's needs. Early intervention and treatment help adolescents overcome addiction, prevent long-term health consequences, and reduce the risk of future substance abuse [27,28].

Collaboration Between Schools, Healthcare Providers, and Community Organizations

To effectively address inhalant abuse and whitener addiction among adolescents, it is crucial to establish collaboration between different stakeholders involved in prevention and treatment efforts. Schools are vital in creating awareness about the dangers of inhalant abuse and whitener addiction among students. They can develop educational programs and support services to promote a healthy lifestyle and provide resources for students struggling with addiction [1,4,8-10,23].

Healthcare providers can assist in the early identification of at-risk individuals and offer appropriate interventions, such as counseling, therapy, or medication. They can also provide support and guidance to families and caregivers unaware of the signs and symptoms of inhalant abuse and whitener addiction [14,22,28].

Community organizations, including nonprofits, faith-based organizations, and local government agencies, can support prevention and treatment efforts by providing resources, funding, and outreach programs. They can collaborate with schools and healthcare providers to offer coordinated support and referral services for individuals struggling with addiction [4,7,15].

Collaboration between these stakeholders can also help to reduce the stigma surrounding addiction and promote a more compassionate and supportive environment for those affected by inhalant abuse and whitener addiction. By working together, they can create a comprehensive approach to address this critical public health issue, ensuring that resources are utilized effectively and strategies are targeted toward the specific needs of the community [24,26,29].

Support for Families Affected by Inhalant Abuse and Addiction

Families affected by inhalant abuse and whitener addiction can face various challenges, including emotional stress, financial strain, and social isolation. These challenges can arise due to the impact of the addiction on family relationships and the costs associated with treatment and recovery. Therefore, providing support and resources to families struggling with these issues is important. Family counseling can help family members better understand the addiction and how it affects their loved one and learn coping and communication strategies. Peer support groups like Al-Anon can provide a sense of community and understanding for families with similar challenges. Additionally, financial assistance can help families to cover the costs of treatment and other expenses related to the addiction. By providing support and resources for families affected by inhalant abuse and whitener addiction, we can help to strengthen family relationships, reduce the impact of the addiction, and support the overall well-being of those affected [5]. Organizations play a crucial role in supporting affected families and mitigating the impact of addiction. Government agencies often offer grants, subsidies, or financial aid programs to assist individuals and families seeking addiction treatment. Nongovernmental organizations, such as charities and foundations, may also provide resources and support programs tailored to those affected by inhalant abuse. Individuals and families should contact local healthcare providers, addiction treatment centers, or community support groups for up-to-date information on available financial assistance options in their respective areas.

## Conclusions

Inhalant abuse and whitener addiction among adolescents pose significant consequences for physical and mental health, families, communities, and society as a whole. To tackle this critical issue, a comprehensive and coordinated approach is required, encompassing education, prevention, early intervention, treatment, collaboration, and support. As policymakers, healthcare providers, educators, parents, and community members, we bear the responsibility of preventing inhalant abuse and whitener addiction among adolescents. To address this problem effectively, it is crucial to consider long-term strategies alongside immediate solutions. Strengthening education and awareness is paramount. Comprehensive education programs should be implemented, focusing on the risks and consequences of inhalant abuse and whitener addiction. Increasing awareness among adolescents, parents, teachers, and communities is vital, ensuring they understand the long-term health effects and potential for addiction associated with these substances. Addressing social and environmental factors is equally important. Recognizing and targeting issues such as peer pressure, parental involvement, substance availability, and underlying mental health conditions through community-based programs is necessary. Creating supportive and drug-free environments for adolescents will contribute to long-term prevention efforts. By incorporating these long-term solutions, we can strengthen our comprehensive and coordinated approach to addressing inhalant abuse and whitener addiction among adolescents. Together, we can raise awareness, provide access to prevention and treatment resources, promote research and innovation, and tackle the social and environmental factors that contribute to substance abuse. Prioritizing the well-being of our youth will enable us to build stronger, healthier communities for generations to come.
